# Conditioned media from dental pulp stem cells improved diabetic polyneuropathy through anti‐inflammatory, neuroprotective and angiogenic actions: Cell‐free regenerative medicine for diabetic polyneuropathy

**DOI:** 10.1111/jdi.13045

**Published:** 2019-04-23

**Authors:** Eriko Makino, Nobuhisa Nakamura, Megumi Miyabe, Mizuho Ito, Saki Kanada, Masaki Hata, Tomokazu Saiki, Kazunori Sango, Hideki Kamiya, Jiro Nakamura, Ken Miyazawa, Shigemi Goto, Tatsuaki Matsubara, Keiko Naruse

**Affiliations:** ^1^ Department of Orthodontics School of Dentistry Aichi Gakuin University Nagoya Japan; ^2^ Department of Internal Medicine School of Dentistry Aichi Gakuin University Nagoya Japan; ^3^ Department of Removable Prosthodontics School of Dentistry Aichi Gakuin University Nagoya Japan; ^4^ Department of Pharmacy Dental Hospital Aichi Gakuin University Nagoya Japan; ^5^ Laboratory of Peripheral Nerve Pathophysiology Tokyo Metropolitan Institute of Medical Science Tokyo Japan; ^6^ Division of Diabetes Department of Internal Medicine Aichi Medical University Nagakute Japan

**Keywords:** Dental pulp stem cell, Diabetic neuropathy, Regenerative medicine

## Abstract

**Aims/Introduction:**

Dental pulp stem cells (DPSCs) can be easily obtained from teeth for general orthodontic reasons. We have previously reported the therapeutic effects of DPSC transplantation for diabetic polyneuropathy. As abundant secretomes from DPSCs are considered to play a central role in the improvement of diabetic polyneuropathy, we investigated whether direct injection of DPSC‐conditioned media (DPSC‐CM) into hindlimb skeletal muscles ameliorates diabetic polyneuropathy in diabetic rats.

**Materials and Methods:**

DPSCs were isolated from the dental pulp of Sprague–Dawley rats. Eight weeks after the induction of diabetes, DPSC‐CM was injected into the unilateral hindlimb skeletal muscles in both normal and diabetic rats. The effects of DPSC‐CM on diabetic polyneuropathy were assessed 4 weeks after DPSC‐CM injection. To confirm the angiogenic effect of DPSC‐CM, the effect of DPSC‐CM on cultured human umbilical vascular endothelial cell proliferation was investigated.

**Results:**

The administration of DPSC‐CM into the hindlimb skeletal muscles significantly ameliorated sciatic motor/sensory nerve conduction velocity, sciatic nerve blood flow and intraepidermal nerve fiber density in the footpads of diabetic rats. We also showed that DPSC‐CM injection significantly increased the capillary density of the skeletal muscles, and suppressed pro‐inflammatory reactions in the sciatic nerves of diabetic rats. Furthermore, an *in vitro* study showed that DPSC‐CM significantly increased the proliferation of umbilical vascular endothelial cells.

**Conclusions:**

We showed that DPSC‐CM injection into hindlimb skeletal muscles has a therapeutic effect on diabetic polyneuropathy through neuroprotective, angiogenic and anti‐inflammatory actions. DPSC‐CM could be a novel cell‐free regenerative medicine treatment for diabetic polyneuropathy.

## Introduction

The onset and progression of diabetic polyneuropathy are fundamentally linked to metabolic disorders and blood flow impediments resulting from chronic hyperglycemia, with immune dysfunction as an additional contributing factor^1^. Painful diabetic neuropathy can now be treated with neurotransmission blockers that produce relatively few adverse reactions, and the options for symptomatic treatment of diabetic polyneuropathy have increased. However, therapeutic strategies targeting the cause of diabetic polyneuropathy are lacking. A particular need exists for radical treatments in cases in which diabetic polyneuropathy has progressed.

Stem cell transplantation is expected to become a novel therapy for diabetic polyneuropathy. We first showed the therapeutic effects of endothelial progenitor cell transplantation for diabetic polyneuropathy^2^. To date, the efficacy of cell transplantation therapy for diabetic polyneuropathy has been reported by us and others using various kinds of stem cells, such as mesenchymal stem cells and embryonic stem/induced pluripotent‐derived cells^3^, ^4^, ^5^, ^6^, ^7^, ^8^. These stem cell transplantations improved nerve conduction velocity, nerve blood flow, intraepidermal nerve fiber density, sensory disorders and nerve morphology.

In contrast, cell dysfunction occurred even in progenitor cells and stem cells under diabetic conditions and aging^9^, ^10^, ^11^, ^12^. The therapeutic efficacy of transplantation of stem cells from aging and/or diabetic animals was impaired compared with that of cells from normal animals^13^, ^14^. To solve these problems, we proposed the use of dental pulp stem cells (DPSCs), a kind of mesenchymal stem cell, for cell transplantation therapy in diabetic polyneuropathy, because DPSCs can be easily isolated from teeth extracted for general orthodontic reasons at young ages, and in many cases before the onset of diabetes, and DPSCs can be cryopreserved until use^15^.

Initially, researchers expected angiogenesis and neuroprotection to occur as a result of local engrafting and differentiation into multiple cell types. However, subsequent studies showed that very few grafted cells survive at the site of transplantation, which was consistent with most cell therapies for other diseases, such as ischemic heart disease and brain infarction^16^, ^17^. Current thinking highlights abundant secretomes from transplanted stem cells, including angiogenic factors, neurotrophic factors and immunosuppressive factors, which might show angiogenic and protective effects at the site of transplantation^18^, ^19^. Therefore, we hypothesized that the administration of DPSC‐secreted factors would be a preferable therapy for diabetic polyneuropathy.

In the present study, we used 10× concentrated DPSC‐conditioned media (DPSC‐CM), and investigated the therapeutic effects of DPSC‐CM on diabetic polyneuropathy. We first showed that DPSC‐CM administration into unilateral hindlimb skeletal muscles significantly ameliorated nerve conduction velocity, nerve blood flow and intraepidermal nerve fiber density. Our results suggest a cell‐free therapeutic strategy in regenerative medicine for diabetic polyneuropathy.

## Methods

### Animals

Male Sprague–Dawley rats were obtained from Chubu Kagakushizai (Nagoya, Japan) at 6 weeks of age. Streptozotocin (Sigma, St. Louis, MO, USA; 60 mg/kg bodyweight in 0.9% sterile saline) was injected intraperitoneally for the induction of diabetes. Rats with a blood glucose level >14 mmol/L were used as diabetic animals. This study was approved by the Institutional Animal Care and Use Committees of Aichi Gakuin University (AGUD318), and all animal experiments were carried out following the national guidelines and the relevant national laws on the protection of animals.

### Culture of DPSCs and the preparation of DPSC‐CM

Six‐week‐old male Sprague–Dawley rats were killed by an overdose of pentobarbital. Dental pulp tissues from the incisors were collected in one vial; DPSCs were isolated and cultured as previously described^15^. Collected dental pulp was suspended in phosphate‐buffered saline containing 0.1% collagenase and 0.25% trypsin‐ethylenediaminetetraacetic acid. DPSCs were cultured in an alpha modification of Eagle's medium (α‐MEM; GIBCO Laboratories Inc., Grand Island, NY, USA), supplemented with 5.5 mmol/L glucose and 20% fetal bovine serum (GIBCO), on plastic dishes in a humidified incubator at 37°C in 5% CO_2_. Non‐adherent cells were washed off, and adherent cells were continuously expanded until passage 3.

To obtain DPSC‐CM, DPSCs were maintained in serum‐free Dulbecco's modified Eagle medium (GIBCO). After 24 h, the culture medium was collected, concentrated by a factor of 10 using 3‐kDa centrifugal filters (Amicon Ultra‐15; Nihon Millipore, Tokyo, Japan) and frozen at −20°C until use.

### Administration of DPSC‐CM

We administered DPSC‐CM into the hindlimb skeletal muscles 8 weeks after streptozotocin injection. DPSC‐CM (1.0 mL/rat) or the same dose of vehicle (Dulbecco's modified Eagle medium) was injected at 10 points in the unilateral hindlimb skeletal muscles of both normal and diabetic rats. Four weeks after administration, the following measurements were carried out.

### Sciatic motor and sensory nerve conduction velocities

After anesthetization by isoflurane, motor nerve conduction velocity (MNCV) between the ankle and the sciatic notch, and sensory nerve conduction velocity (SNCV) between the ankle and the knee were assessed using a Neuropak MEB‐9400 instrument (Nihon‐Koden, Osaka, Japan). Rats were placed on a warming pad to maintain the near‐nerve temperature at 37°C, monitored by a BAT‐12 multipurpose thermometer (Bioresearch Co., Nagoya, Japan).

### Sciatic nerve blood flow

Rats were placed on a warming pad and anesthetized with isoflurane. The femur skin was cut, and a laser probe was placed just above the exposed sciatic nerve. Sciatic nerve blood flow (SNBF) was measured using a Laser Doppler Blood Flow Meter (FLO‐N1; Omega Wave Inc., Tokyo, Japan).

### Intraepidermal nerve fiber density of the plantar skins

After fixation, footpads were immersed in an optimal cutting temperature compound (Sakura Finetechnical, Tokyo, Japan). Then, 25‐μm thick footpad sections were cut on a cryostat. The sections were incubated with anti‐PGP9.5 antibody (Millipore). Alexa Fluor 594‐coupled goat anti‐mouse immunoglobulin G antibody (Invitrogen, Carlsbad, CA, USA) was applied as the secondary antibody. Nerve fibers were counted under an FV10i confocal system (Olympus, Tokyo, Japan).

### Immunohistological staining

Paraffin‐embedded sciatic nerves and gastrocnemius muscles were cut into 5‐μm sections for immunohistochemical staining. The nerve sections were incubated with anti‐CD68 polyclonal antibody (Abcam, Cambridge, UK) or anti‐platelet endothelial cell adhesion molecule 1 (PECAM‐1) monoclonal antibody (Dianova, Hamburg, Germany) and subsequently stained using the Simplestain rat system (Nichirei, Tokyo, Japan) according to the manufacturer's instructions. The muscle sections were incubated with anti‐PECAM‐1 monoclonal antibody and stained using an Alexa Fluor 594‐coupled goat anti‐mouse immunoglobulin G antibody (Invitrogen). Slides were observed under light and fluorescence microscope.

### Messenger ribonucleic acid expression in sciatic nerves and hindlimb skeletal muscles

Tissues were immersed in RNAlater ribonucleic acid stabilization reagent (Qiagen, Valencia, CA, USA) and extracted using an RNeasy Mini Kit (Qiagen) according to the manufacturer's instructions. Complementary deoxyribonucleic acid was synthesized using ReverTra Ace (Toyobo, Osaka, Japan). TaqMan Gene Expression Assay primers and probes for tumor necrosis factor (TNF)‐α (*tnf*), CD68 (*CD68*), vascular endothelial growth factor (VEGF; *vegf*) and basic fibroblast growth factor (bFGF; *fgf2*) were purchased from Applied Biosystems (Foster City, CA, USA). Real‐time quantitative polymerase chain reaction was carried out and measured with an ABI Prism 7000 (Applied Biosystems). Relative quantity was calculated with the ΔΔCt method using β_2_ microglobulin as the endogenous control.

### Cell proliferation assay in human umbilical vein endothelial cells

Human umbilical vein endothelial cells (HUVECs) were obtained from the American Type Culture Collection (ATCC, Manassas, VA, USA). HUVECs were cultured in EGM‐2 MV complete medium (LONZA; Walkersville, MD, USA). When HUVECs reached 50% confluence in 24‐well dishes, the cells were starved in serum‐free endothelial cell growth medium 2. After starvation, cells were incubated with DPSC‐CM and 3‐(4,5‐di‐methylthiazol‐2‐yl)‐2,5‐diphenyltetrazolium bromide (MTT; Sigma). After a 2‐h incubation, HUVECs were lysed, and the absorbance at 550 nm was read using a spectrophotometer (Spark; TECAN, Männedorf, Switzerland).

Another cell proliferation assay was carried out using a Cell Counting Kit‐8 (CCK‐8; Dojindo, Kumamoto, Japan) according to the manufacturer's procedure. After starvation, cells were incubated with DPSC‐CM and CCK‐8 for 2 h. For each well, the absorbance at 450 nm was read on a spectrophotometer.

### Statistical analysis

All group values are expressed as the mean ± standard error of the mean. Statistical analysis was carried out using one‐way anova with the Bonferroni correction for multiple comparisons. Differences were considered significant at *P *<* *0.05.

## Result

### Bodyweights and blood glucose levels

The diabetic rats showed severe hyperglycemia (vehicle‐injected normal rats 4.8 ± 0.7 mmol/L, vehicle‐injected diabetic rats 23.2 ± 7.2 mmol/L; *P *<* *0.01) and significantly reduced bodyweight (vehicle‐injected normal rats 435.0 ± 17.3 g, vehicle‐injected diabetic rats 245.0 ± 56.9 g; *P *<* *0.01). Normal and diabetic rats administered DPSC‐CM did not show significant changes in blood glucose or bodyweight compared with vehicle‐administered normal and diabetic rats (blood glucose: DPSC‐CM‐injected normal rats 4.7 ± 0.5 mmol/L, DPSC‐CM‐injected diabetic rats 20.3 ± 4.8 mmol/L; bodyweight: DPSC‐CM‐injected normal rats 445.0 ± 34.2 g, DPSC‐CM‐injected diabetic rats 214.0 ± 25.1 g).

### DPSC‐CM improved MNCV, SNCV and SNBF in the diabetic rats

**Figure 1:**
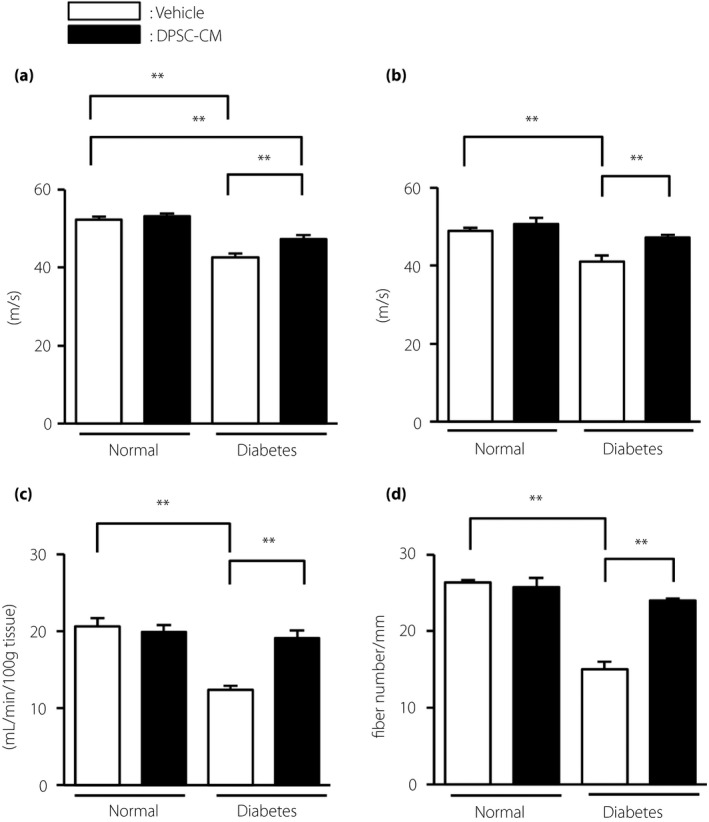
The administration of dental pulp stem cell‐conditioned media (DPSC‐CM) improved the delay in sciatic nerve conduction velocities, and the decrease in sciatic nerve blood flow and intraepidermal nerve fiber density in diabetic rats. (a) Sciatic nerve motor nerve conduction velocities. Motor nerve conduction velocity was measured between the ankle and sciatic notch (*n *=* *5–7). (b) Sciatic nerve sensory nerve conduction velocities. Sensory nerve conduction velocity was measured between the ankle and knee (*n *=* *5–7). (c) Sciatic nerve blood flow. Sciatic nerve blood flow was measured using a laser Doppler blood flow meter (*n *=* *5–7). (d) Intraepidermal nerve fiber density of the footpads (*n* = 4). The results are presented as the mean ± standard error of the mean. **P <* *0.01. Measurements were carried out 4 weeks after the DPSC‐CM injection.

Four weeks after injection with DPSC‐CM, MNCV, SNCV and SNBF were measured. The vehicle‐injected diabetic rats showed significantly delayed MNCV and SNCV, and decreased SNBF compared with the vehicle‐injected normal rats (Figure 1a,b,c; *P *<* *0.01). Notably, DPSC‐CM injection significantly increased MNCV, SNCV and SNBF in the diabetic rats (*P *<* *0.01). In contrast, the administration of DPSC‐DM to normal rats did not affect MNCV, SNCV or SNBF.

When we compared these parameters in diabetic rats injected with DPSC‐CM on one side and vehicle on the opposite side, MNCV and SNCV were significantly improved in the DPSC‐CM‐injected side compared with those in the opposite side (Figure S1). SNBF tended to be increased on the DPSC‐CM‐injected side, but this difference was not significant.

### DPSC‐CM increased intraepidermal nerve fiber density of the footpads in diabetic rats

Quantitative analyses revealed that the vehicle‐injected diabetic rats showed significantly reduced intraepidermal nerve fiber density (IENFD) compared with the vehicle‐injected normal rats (vehicle‐injected normal rats: 26.3 ± 0.3/mm, vehicle‐injected diabetic rats: 15.0 ± 1.0/mm; *P *<* *0.01; Figure 1d). DPSC‐CM administration significantly increased IENFD in the diabetic rats (24.0 ± 0.2 /mm; *P *<* *0.01), although it did not affect IENFD in the normal rats.

### DPSC‐CM suppressed inflammation in the sciatic nerves of diabetic rats

**Figure 2:**
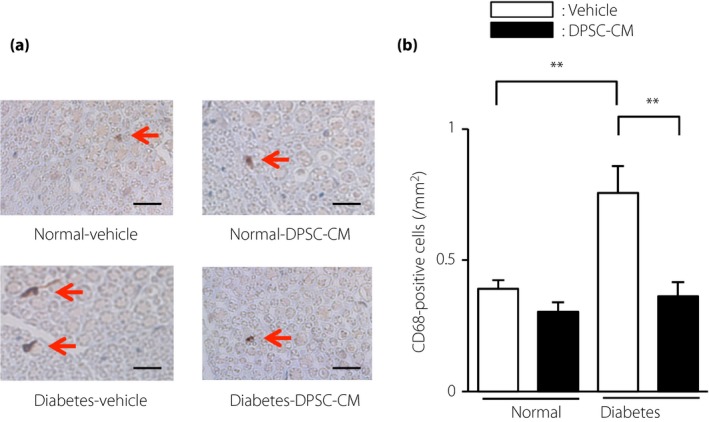
Dental pulp stem cell‐conditioned media (DPSC‐CM) injection suppressed the number of CD68‐positive macrophages in sciatic nerves of diabetic rats. (a) Representative photomicrographs of the sciatic nerves of normal and diabetic rats injected with vehicle or DPSC‐CM. Macrophages were detected by immunostaining for CD68. Scale bar, 10 μm. (b) Quantitative analyses of CD68‐positive cells/mm^2^ in the sciatic nerves of normal and diabetic rats (*n *=* *4). The results are presented as the mean ± standard error of the mean. **P <* *0.01.

The number of macrophages in the sciatic nerves was visualized by staining with CD68 antibody (Figure 2a). The number of macrophages was significantly increased in the diabetic rats (vehicle‐injected diabetic rats: 0.80 ± 0.10/mm^2^, vehicle‐injected normal rats: 0.40 ± 0.03/mm^2^, *P *<* *0.01; Figure 2b). Furthermore, the administration of DPSC‐CM to the diabetic rats significantly suppressed the number of macrophages in the sciatic nerves (0.40 ± 0.05/mm^2^, *P *<* *0.01). However, no significant difference was observed between the vehicle‐injected and DPSC‐CM‐injected normal rats.

**Figure 3:**
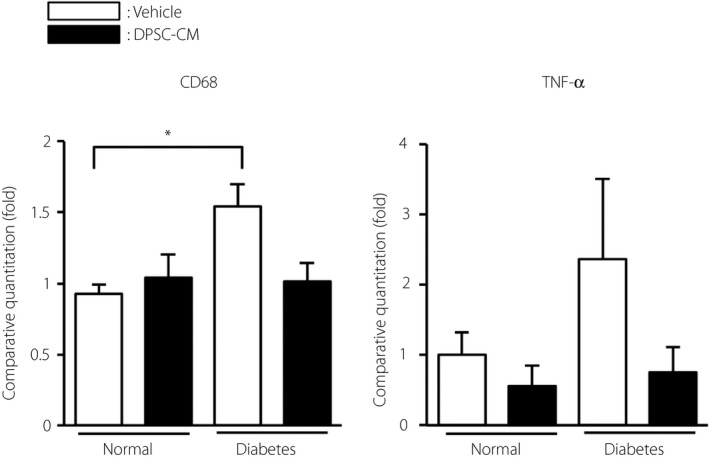
The effects of dental pulp stem cell‐conditioned media (DPSC‐CM) on the inflammatory messenger ribonucleic acid expressions in sciatic nerves of normal and diabetic rats. Four weeks after the injection of DPSC‐CM, the messenger ribonucleic acid expression of CD68 (*CD68*) and tumor necrosis factor (TNF‐α; *Tnf*) in the sciatic nerves was evaluated by real‐time quantitative polymerase chain reaction (*n *=* *4–6). The results are presented as the mean ± standard error of the mean. *P <* *0.05.

We confirmed that the gene expression of CD68 was significantly increased in the sciatic nerves of diabetic rats (*P *<* *0.05), and was ameliorated by the administration of DPSC‐CM (Figure 3). An increasing trend of expression of the pro‐inflammatory gene, TNF‐α, was also seen in the sciatic nerves of diabetic rats, although this increase was not significant. DPSC‐CM did not affect CD68 and TNF‐α gene expression in the normal rats.

### Number of capillaries in sciatic nerves was unaffected by DPSC‐CM

**Figure 4:**
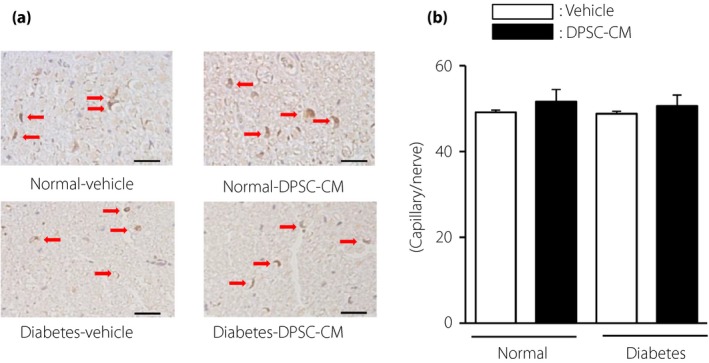
The number of capillaries in sciatic nerves was unaffected by dental pulp stem cell‐conditioned media (DPSC‐CM). (a) Representative photomicrographs of immunohistological staining of the sciatic nerves of normal and diabetic rats. Capillaries were visualized with platelet endothelial cell adhesion molecule 1. Scale bar, 10 μm. (b) Quantitative analysis of the number of capillaries in sciatic nerves of normal and diabetic rats (*n *=* *4). The results are presented as the mean ± standard error of the mean.

The capillaries were visualized by staining with PECAM‐1 antibody (Figure 4a). The number of capillaries in sciatic nerves was similar in the normal and diabetic rats (vehicle‐injected normal rats: 49.2 ± 0.5, vehicle‐injected diabetic rats: 48.8 ± 0.6; Figure 4b). The administration of DPSC‐CM into the hindlimb skeletal muscles did not affect the capillary number in sciatic nerves (DPSC‐CM‐injected normal rats: 51.6 ± 2.9, DPSC‐CM‐injected diabetic rats: 50.6 ± 2.6).

### DPSC‐CM increased the capillary density of the hindlimb skeletal muscles in diabetic rats

**Figure 5:**
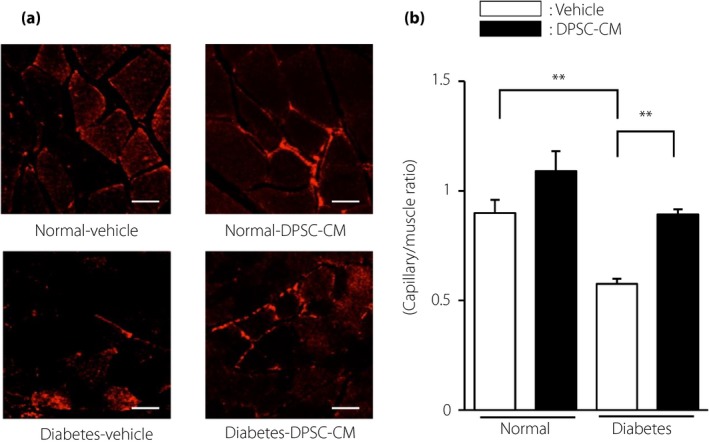
The administration of dental pulp stem cell‐conditioned media (DPSC‐CM) increased the capillary density in the hindlimb skeletal muscles of diabetic rats. (a) Representative photomicrographs of immunohistological staining of the skeletal muscles of normal and diabetic rats. Capillaries were visualized with platelet endothelial cell adhesion molecule 1. Scale bar, 50 μm. (b) Quantitative analysis of the capillary‐to‐muscle fiber ratio of the skeletal muscles of normal and diabetic rats (*n *=* *4). The results are presented as the mean ± standard error of the mean. **P <* *0.01.

Capillary density in the hindlimb skeletal muscles was significantly decreased in the diabetic rats (vehicle‐injected diabetic rats: 0.58 ± 0.02, vehicle‐injected normal rats: 0.90 ± 0.06 capillary number/muscle fiber, *P *<* *0.01; Figure 5a,b). Interestingly, DPSC‐CM significantly increased the capillary‐to‐muscle ratio in diabetic rats (DPSC‐CM‐injected diabetic rats: 0.90 ± 0.02 capillary number‐to‐muscle fiber, *P *<* *0.01). The capillary‐to‐muscle ratio in the normal rats was not affected by DPSC‐CM.

### Impact of DPSC‐CM administration on VEGF and bFGF gene expression in the hindlimb skeletal muscles

**Figure 6:**
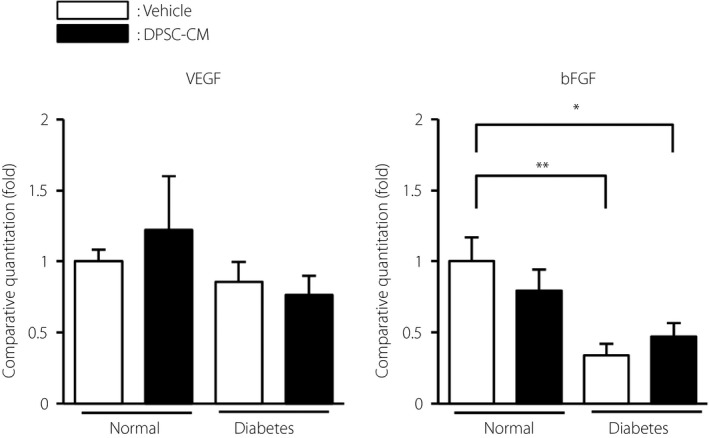
Effects of dental pulp stem cell‐conditioned media (DPSC‐CM) on messenger ribonucleic acid expression in the hindlimb skeletal muscles. Four weeks after injection with DPSC‐CM, the messenger ribonucleic acid expression levels of vascular endothelial growth factor (VEGF) and basic fibroblast growth factor (bFGF) in the hindlimb skeletal muscles were evaluated by real‐time quantitative polymerase chain reaction. The results are presented as the mean ± standard error of the mean (*n *=* *4–7). *P <* *0.05, **P <* *0.01.

No significant difference in VEGF gene expression in the hindlimb skeletal muscles was found between the normal and diabetic rats (Figure 6). In contrast, the gene expression levels of bFGF were significantly decreased in the diabetic rats compared with the normal rats (vehicle‐injected diabetic rats: 0.34 ± 0.08 vs vehicle‐injected normal rats, *P *<* *0.01). DPSC‐CM did not change VEGF or bFGF gene expression in the hindlimb skeletal muscles at 4 weeks after injection in either normal or diabetic rats.

### DPSC‐CM increased the proliferation of vascular endothelial cells

**Figure 7:**
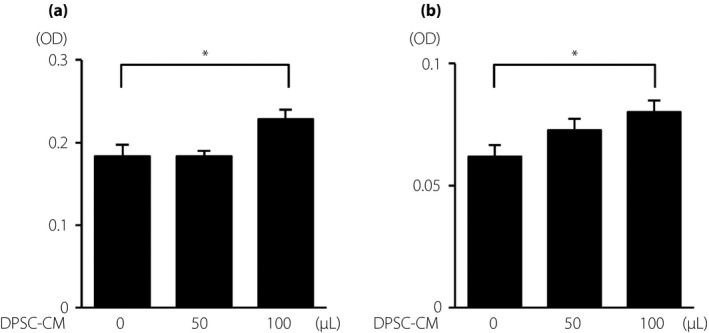
Dental pulp stem cell‐conditioned media (DPSC‐CM) promoted vascular endothelial cell viability. (a) Cell proliferation was assessed by cell counting kit‐8. (b) Cell proliferation was assessed with a 3‐(4,5‐di‐methylthiazol‐2‐yl)‐2,5‐diphenyltetrazolium bromide assay. The results are presented as the mean ± standard error of the mean (*n *=* *8). *P <* *0.05.

We investigated whether DPSC‐CM directly increased the proliferation of HUVECs. Cell proliferation was assessed by CCK‐8 and MTT assays. DPSC‐CM significantly increased the proliferation of HUVECs, as shown by a 1.2‐fold increase by CCK‐8 assay (Figure 7a) and a 1.3‐fold increase by the MTT assay (Figure 7b; *P *<* *0.05).

## Discussion

In the present study, we first showed the therapeutic efficacy of DPSC‐CM on diabetic polyneuropathy. Administration of DPSC‐CM into the hindlimb skeletal muscles significantly ameliorated sciatic MNCV, SNCV and SNBF, as well as IENFD in the footpads of diabetic rats. We also showed that DPSC‐CM injection significantly increased the capillary density of hindlimb skeletal muscles and suppressed the pro‐inflammatory reaction in sciatic nerves of diabetic rats.

We have previously reported the therapeutic effect of DPSC transplantation on diabetic polyneuropathy^15^, ^20^, ^21^. However, many of the engrafted DPSCs disappeared from the transplanted site during a certain period after transplantation, which is consistent with other cell transplantation therapies for ischemic heart disease and brain infarction^17^, ^22^. Therefore, we hypothesized that the therapeutic mechanisms of stem cell transplantation were mainly associated with the abundant secretomes of transplanted stem cells in the early phase of transplantation. In the present study, a single injection of DPSC‐CM into the hindlimb skeletal muscles ameliorated diabetic polyneuropathy by promoting the recovery of sciatic MNCV and SNCV, and the increase in IENFD of diabetic rats. Furthermore, DPSC‐CM administration reduced the number of macrophages in the sciatic nerves of diabetic rats.

When we compare the therapeutic effects between DPSC‐CM administration in the present study and DPSC transplantation in our previous study, the effects of DPSC‐CM administration on SNCV and SNBF were comparable to those of DPSC transplantation^15^. However, the effect of DPSC‐CM for MNCV was lower than that of DPSC transplantation (data not shown). We will investigate the doses and timing of DPSC‐CM administration to improve efficacy in the future.

We previously confirmed the gene expression of angiogenic factors, neurotrophic factors and immunomodulatory factors in cultured DPSCs^20^. Under inflammatory conditions, DPSC‐CM increased the anti‐inflammatory marker genes, CD206 and interleukin‐10, in macrophages and induced M2 polarization^20^. In neuronal cells, DPSC‐CM elongated neurite outgrowth of dorsal root ganglion cells, and increased the proliferation and myelin formation of Schwann cells^21^.

A previous study showed decreased capillary density in skeletal muscles in diabetic patients^23^. We showed that DPSC‐CM improved the decrease in capillary density in the skeletal muscles of diabetic rats. This phenomenon might be due to the direct angiogenic effects of DPSC‐CM, as DPSC‐CM contains abundant VEGF^21^ and significantly increases the viability of HUVECs in culture. However, the direct relationship between capillary density in the skeletal muscles and diabetic neuropathy is still unclear. Just a few studies have investigated the relationship between capillary density in skeletal muscles and diabetic neuropathy. Andreassen *et al*.^24^ showed no relationship between capillary density and the degree of neuropathy. However, the sample size was not large enough in that study.

We observed the decrease of nerve blood flow in the diabetic rats, which was ameliorated by DPSC‐CM administration. In contrast, the capillary number in the sciatic nerves was similar between the normal and diabetic rats, and DPSC‐CM administration did not affect the capillary number in sciatic nerves. As the major morphological changes in the capillaries in the peripheral nerves of diabetes patients are basement membrane thickness, vascular endothelial cell hyperplasia and reduction in lumen area^25^, ^26^, DPSC‐CM might affect them. Further study is required in this field.

Because DPSC‐CM contains VEGF, we must consider the risk of diabetic retinopathy. Thus far, the risk of worsening diabetic retinopathy by DPSC‐CM administration to the lower limb does not seem high, because a clinical trial for diabetic polyneuropathy showed that VEGF gene transfer to the legs did not show worse proliferative retinopathy^27^. However, selecting patients without proliferative retinopathy and monitoring diabetic retinopathy in the case of DPSC‐CM administration might be necessary to avoid the risk of worsening diabetic retinopathy.

The therapeutic effects of mesenchymal stem cell‐conditioned media have been explored in other diseases, such as ischemic heart disease^28^, brain injury^29^, spinal cord injury^30^ and bone defects^31^. The use of cell‐free therapies, such as conditioned media from mesenchymal stem cells, has specific advantages over stem cell‐based therapies. First, conditioned media was free from graft versus host disease, tumorigenicity and embolus formation^32^. Second, large‐scale production of conditioned media reduces the cost and maintains the high quality of conditioned media. Third, treatment for acute conditions, such as ischemic heart disease and brain infarction, is immediately available. In addition, the advantage of DPSC‐CM is that we can continuously obtain young and high‐quality DPSCs from teeth extracted for general orthodontic reasons at young ages and, in many cases, before the onset of several diseases without further invasion.

We used 10‐fold concentrated DPSC‐CM using a 3‐kDa filter for protein. Because the main proteins of the angiogenic, neurotrophic and immunomodulated factors are larger than 3‐kDa (e.g., VEGF, bFGF, nerve growth factor [NGF], neurotrophin [NT]‐3 and macrophage colony‐stimulating factor; gene expression of all of these has been confirmed in DPSCs^20^), this method is useful for reducing the administration volume without loss of efficacy. The combination of angiogenic, neurotrophic and immunomodulatory effects might show multifocal improvement of diabetic polyneuropathy. VEGF gene transfer to the lower limbs increased vascularity and improved symptoms of diabetic polyneuropathy^27^, ^33^. bFGF has angiogenic and neurotrophic effects, and we previously showed that intramuscular administration of bFGF with cross‐linked gelatin hydrogel improved SNCV and SNBF in diabetic rats^34^. Neurotrophic factors, such as NGF and NT‐3, are reduced in diabetic animals^35^, ^36^. NGF binds to p75 and tropomyosin receptor kinase A with effects on small sensory and autonomic nerve fibers. In contrast, NT‐3 binds to tropomyosin receptor kinase C, which is expressed in the large fibers. Although clinical trials with NGF were unsuccessful, many animal studies showed the prevention of pain sensation reduction^37^, ^38^. NT‐3 prevented abnormalities in neurofilament biology and mitochondrial dysfunction in diabetic animals^39^, ^40^. Macrophage colony‐stimulating factor promoted macrophage polarization toward the anti‐inflammatory M2 type^41^. Furthermore, macrophage migration and activation were increased in the sciatic nerves in diabetic rats^42^, ^43^. Our previous study confirmed that DPSC‐CM promoted M2 polarization in lipopolysaccharide‐stimulated RAW264.7 cells^20^.

We used a single injection of DPSC‐CM, and carried out an analysis 4 weeks post‐injection in the present study. However, we need to further investigate the doses and timing of DPSC‐CM injections, as well as the duration of efficacy in future experiments.

In conclusion, we showed that DPSC‐CM injection into hindlimb skeletal muscles has therapeutic effects on diabetic polyneuropathy through neuroprotective, angiogenic and anti‐inflammatory actions. In comparison with stem cell transplantation, the use of DPSC‐CM will lead to a reduction in medical costs, maintenance of DPSC‐CM quality by using selected DPSCs with high viability, and freedom from immune incompatibility and tumorigenicity. These advantages will extend DPSC‐CM injection therapy as a treatment for diabetic polyneuropathy.

## Disclosure

The authors declare no conflict of interest.

## Supporting information


**Figure S1** | The comparison of neurophysiological parameters between the dental pulp stem cell‐conditioned media (DPSC‐CM)‐injected side and vehicle‐injected opposite side in the diabetic rats.
