# Supplementary figures and images for: Conditioned media from dental pulp stem cells improved diabetic polyneuropathy through anti‐inflammatory, neuroprotective and angiogenic actions: Cell‐free regenerative medicine for diabetic polyneuropathy
Source: J Diabetes Investig. 2019 Apr 23;10(5):1199–208. doi: 10.1111/jdi.13045 (PMC6717901; doi:10.1111/jdi.13045)

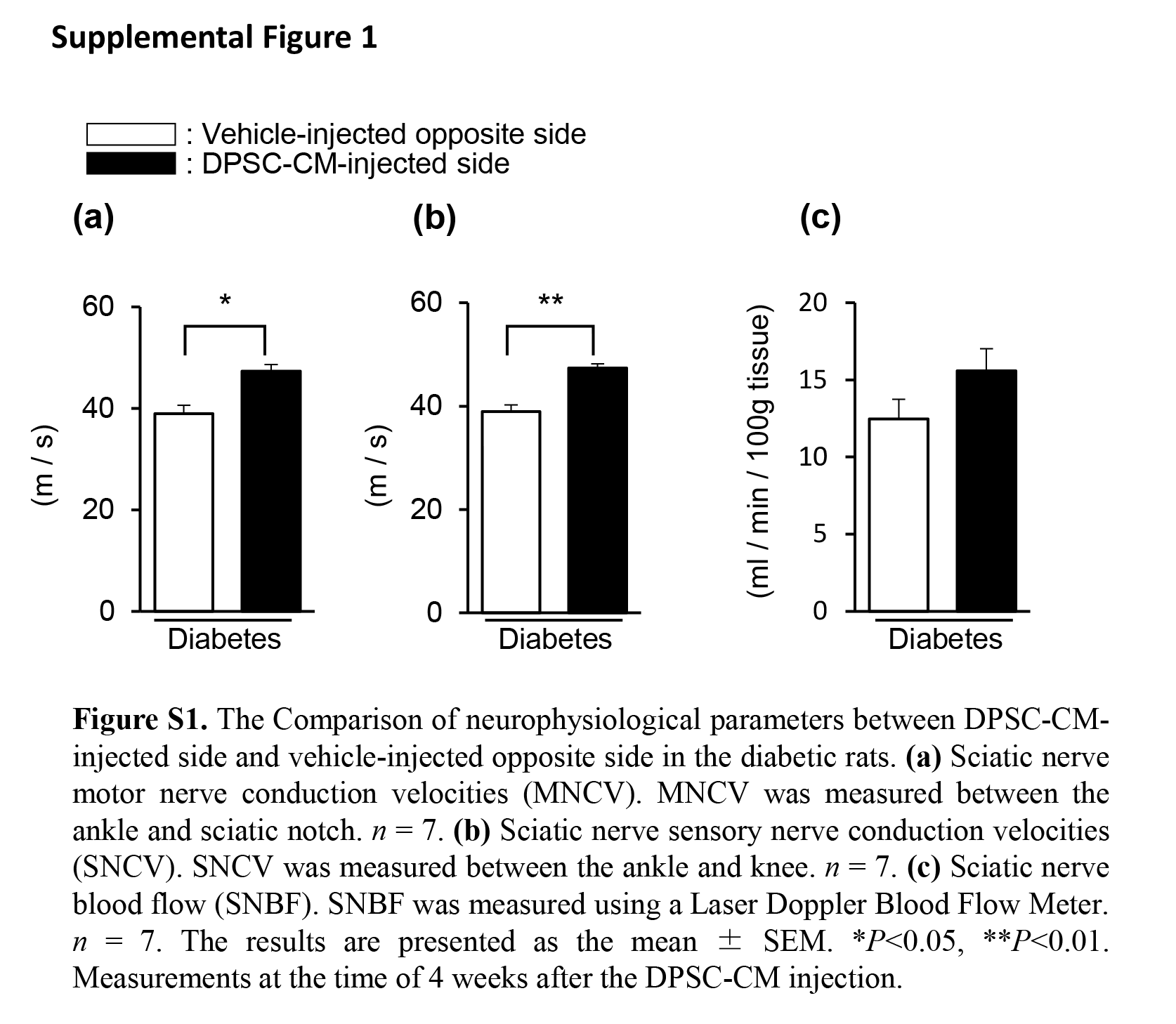

Supplement: Supplementary file 1 — Figure S1 | The comparison of neurophysiological parameters between the dental pulp stem cell‐conditioned media (DPSC‐CM)‐injected side and vehicle‐injected opposite side in the diabetic rats. [file JDI-10-1199-s001.tif]
